# An exploratory analysis of sociodemographic characteristics with ultrafine particle concentrations in Boston, MA

**DOI:** 10.1371/journal.pone.0263434

**Published:** 2022-03-30

**Authors:** Katherine L. Thayer, Kevin Lane, Matthew C. Simon, Doug Brugge, Christina H. Fuller

**Affiliations:** 1 Tufts Medical Center, Boston, MA, United States of America; 2 Tufts University School of Medicine, Boston, MA, United States of America; 3 Boston University School of Public Health, Boston, MA, United States of America; 4 U.S. Department of Transportation, Volpe National Transportation Systems Center, Cambridge, MA, United States of America; 5 University of Connecticut Department of Public Health Sciences, Farmington, CT, United States of America; 6 Georgia State University School of Public Health, Atlanta, GA, United States of America; University of Cape Coast, GHANA

## Abstract

Little is known of the relationship between exposure to the smallest particles of air pollution and socio-demographic characteristics. This paper explores linkages between ultrafine particle (UFP) concentrations and indicators of both race/ethnicity and socioeconomic status in Boston, Massachusetts, USA. We used estimates of UFP based on a highly-resolved land-use regression model of concentrations. In multivariate linear regression models census block groups with high proportions of Asians were associated with higher levels of UFP in comparison to block groups with majority White or other minority groups. Lower UFP concentrations were associated with higher homeownership (indicating higher SES) and with higher female head of household (indicating lower socioeconomic status). One explanation for the results include the proximity of specific groups to traffic corridors that are the main sources of UFP in Boston. Additional studies, especially at higher geographic resolution, are needed in Boston and other major cities to better characterize UFP concentrations by sociodemographic factors.

## Introduction

Particulate matter (PM) air pollution is a significant public health problem and major contributor to the global burden of disease [1]. PM is characterized by size from the largest particles, greater than 10 microns in aerodynamic diameter, to the smallest particles, less than 0.1 microns in aerodynamic diameter (ultrafine particles [UFP]). Particle size contributes to the spatial and temporal distribution of PM and as well as deposition in the lung, transport within the body and health effects [2]. Fine PM (PM_2.5_, less than 2.5 microns in aerodynamic diameter) is a pollutant that is well established as a health concern and is regulated in the United States and other countries [3]. However, there is emerging literature indicating the importance of UFP, which are currently unregulated and have been shown to be in high concentrations near traffic sources [4]. In fact, in most urban areas the predominant source of UFP is traffic pollution [5].

Increased prevalence of cardiovascular and respiratory disease; lung cancer; neurological deficits; and low birth weight have been established for people living near busy roadways. These outcomes are likely to be at least partly linked to local elevations in UFP number rather than to PM_2.5_ mass [6]. UFP may present an additional health risk beyond that posed by PM_2.5_, because its spatiotemporal variation differs from fine PM. UFP have been shown to have steep concentration gradients in and near Boston, Massachusetts (USA) highways, while PM_2.5_ concentrations are largely flat [7]. In addition, studies have shown that UFP can be deposited in the lung and have the ability to translocate to other parts of the body such as the brain [5,8,9]. Given the substantial variation in local concentrations some populations are expected to have greater exposure than others, possibly resulting in an inequitable burden.

A disproportionate share of hazardous air pollution exposure falls on communities of color and low-income communities, which whether intentional or not, contributes to environmental injustice [10]. Achieving environmental justice (EJ) requires bringing about equity in the distribution of environmental hazards and benefits. EJ principles emerged from multiple struggles, most notably the U.S. Civil Rights movement [11,12]. Communities of color, low-income, poor and immigrant neighborhoods have in the past and continue to be targeted for the placement of industrial facilities such as chemical plants, oil refineries and hazardous waste landfills [11,13]. In addition, many of these communities are disproportionately impacted by highways, transportation hubs and railroads and the pollution that they bring [10,14]. A major focus of EJ epidemiology is to identify and document existing inequities in environmental exposures and disease risks. Studies rely on metrics that estimate socioeconomic status (SES) such as individual measures (like income) and indices (like the Synthetic Deprivation Index). Past research has shown that neighborhoods with higher racial/ethnic minority populations as well as those of lower SES experience higher air pollution exposure [15–18]. In particular, spatial analyses have shown that busy roadways are more often located close to minority populations and communities of lower SES [19,20]. However, research on inequitable exposure to particulate air pollution has focused almost exclusively on PM_2.5_ and PM_10_ (< 10 microns in aerodynamic diameter) to date [21].

A recent global review of SES found consistent results in the United States, Africa, Asia and New Zealand, showing that lower SES was linked to higher exposure to multiple pollutants, including PM_2.5_ and PM_10_ [22]. Research from Europe has reported positive associations in some studies and inverse associations in others. To our knowledge there is no review examining race/ethnicity and air pollution globally [22]. In the United States, evidence shows a persistent inequity whereby Black, Asian, Hispanic/Latino and lower SES populations have higher air pollution compared to predominantly White and wealthier areas [15].

The existing weight of evidence is that exposure to UFP concentrations pose an independent risk to health compared to larger size fractions due to their distinct size and spatiotemporal variation in ambient air [21]. Therefore, attention to only larger size fractions is insufficient to address adverse impacts from particulate matter as a whole. Identifying characteristics and factors putting specific populations at greatest risk for exposure is imperative to understand and later remedy excess burdens. Actions to address disproportionate exposures to PM_2.5_ and PM_10_ may not remedy UFP exposures and associated health risks, because associations between sociodemographic factors and PM_2.5_ and PM_10_ may be similar to or different from those with UFP. In addition, the analyses in this paper begin to address some of these knowledge gaps. We analyzed UFP concentrations (i.e., particle number concentration, or PNC, a proxy for UFP) across a large part of the City of Boston, Massachusetts (USA) and tested their association with indicators of SES and racial/ethnic composition.

## Methods

### Geographic context

Boston is a city of roughly 700,000 people situated on the Atlantic coast of the United States in eastern Massachusetts. The downtown area (and primary business district) of the city is located next to the water with the majority of inhabitants residing outside this area. Boston is crossed by several highways including Interstate-93 (I-93) running north to south through the city as well as Interstate-90 (I-90), which runs east to west. The highways combined carry approximately 300,000 vehicles through the city each weekday. The city has a large public transit system that incorporates subways, buses, trolleys and commuter rail. The vast road network in the downtown area is congested with both local and regional travel. The city is also home to a seaport and major international airport.

Boston ranks among the top 20% of the most segregated cities in the United States [23]. Racial/ethnic minorities are concentrated in the southern part of Boston, with the highest concentration in south-central Boston where they compose 90–100% of the population. Neighborhoods where Whites are the majority include downtown Boston along with the eastern and western parts of the city. (Refer to S1 Fig for a map of city neighborhoods.) More specifically, in evaluating race and ethnicity by census tracts and neighborhoods we find that there is the greatest degree of segregation is between Black and White Bostonians. Majority-Black-resident census tracts are located in the neighborhoods of Roxbury, Dorchester and Mattapan, which are 41%, 45% and 80% Black, respectively. While the majority-White-resident census tracts are concentrated in the neighborhoods such as Back Bay, Charlestown and South Boston, which are 78%, 76% and 79% White, respectively. The Chinatown neighborhood (located in the Downtown area) has the highest proportion of Asians (Chinese and Chinese-American) at 21% compared to 45% White. The community of East Boston is 53% Hispanic/Latino.

Neighborhoods with the highest incomes are located in the northern and western parts of the city including Back Bay, Beacon Hill, South Boston, Jamaica Plain, and Hyde Park [23]. Lower income residents are concentrated in the southern-central neighborhoods of Roxbury, Dorchester and Mattapan and the eastern neighborhood of East Boston. Highest poverty is found in Roxbury and Dorchester, with percentages above 30%, and Allston/Brighton where the poverty rate is 25%.

### Estimation of UFP concentrations

UFP concentrations were estimated using a recently developed highly-resolved land-use regression (LUR) model for the Boston area (Simon et al., 2018). The LUR model estimates the spatial variation of UFP throughout the study area at a 20 x 20 m resolution using spatially-resolved predictors (i.e., distance from major roadways, distance from different land uses, and distance from open spaces). A complete description of model development and assessment is described in Simon et al (2018). Briefly, UFP concentrations were measured via mobile monitoring along a fixed route in Boston (between 05:00 and 22:00; in all seasons; on all days of the week) and by continuously monitoring at a stationary reference site (24 hr/day, 7 days/week) near the center of the study area. Data were collected from December 2011 to November 2013. To develop the LUR model, spatial covariates were regressed against 1-second UFP measurements made while mobile monitoring after first normalizing the mobile-monitoring measurements by the reference-site concentrations. This had the effect of removing much of the temporal dependency in the UFP data set. The resulting model estimates the location-specific deviation of UFP (i.e., spatial factor) relative to the reference site. The model was then used to estimate the spatial variability of UFP across the study area relative to the annual average UFP measured at the reference site.

We multiplied the annual average reference-site UFP (15,000 particles/cm^3^ in Boston in 2013) by each of the 20 x 20 m grid cells of spatial factors to estimate annual average UFP concentrations at the same spatial resolution. This modeling framework–pairing an LUR model with measured UFP data at a reference site–was shown to perform moderately well when compared to an external dataset of 20 residential sites. Models had a Pearson correlation coefficient of 0.74 in Boston for UFP modeled at an hourly resolution [24]. The 20 x 20 m grid was superimposed over a map of block groups in Boston obtained from 2010 US Census TIGER Shapefiles using ArcMap 10.5.1. The grids were then pooled using a spatial join based on mean calculation of exposure at each square’s centroid to calculate the average annual UFP concentration for each block group. Along borders, grids that overlapped with the block groups by 50% or more were included.

### Census socioeconomic and demographic data

Demographic data were obtained from the American Community Survey (ACS) 3-year estimates for 2013 at the block group level. Based on the literature and availability of data, the following variables were chosen as SES indicators in this study: poverty, male unemployment, education, public assistance, home ownership, female-headed households (female HOH), median income (Table 1). There is prior evidence for a direct association between these variables, except for home ownership and median income for which there is evidence of an inverse association. We also included racial and ethnic categories, specifically, non-Hispanic/Latino Black (Black); non-Hispanic/Latino Asian (Asian); White; and Hispanic/Latino of any race (Hispanic/Latino) [25–27]. Any census block groups with values of 0 were excluded from the analysis.

**Table 1:** List of variables, definitions and descriptive statistics among block groups in the study area (n = 250).

Variable Name	Definition^1^	Mean (min, max)	SD
UFP^3^	2013 Mean Annual Ambient UFP (particles/cm^3^) by block group	25,000 (10,385, 37,666)	5,000
Poverty	% of total households in a block group whose median income in the past 12 months was below the poverty level	26.6 (0.0, 100.0)	17.8
Male Unemployment	% of males aged 16–64 in a block group who were unemployed	22.7 (0.0, 88.1)	15.4
Education	% of adults aged ≥ 25 in a block group who have less than a high school education	15.3 (0.0, 65.5)	13.6
Public Assistance	% of households in a block group receiving public assistance	5.2 (0.0, 32.9)	6.5
Home Ownership	% of occupied households that are owned by people who are living in them	30.0 (0.0, 100.0)	21.7
Female Headed Households	% of households with female heads of household (no husband present) and at least one resident under the age of 18	12.7 (0.0, 68.0)	13.9
Median Income^2^	Median household income over past 12 months	$57,000 ($30,000, $250,000)	4.66
Race and Ethnicity			
Black	% of population that self-identifies as Black, non-Hispanic/Latino	23.11 (0.00, 94.76)	25.16
Asian	% of population that self-identifies as Asian, non-Hispanic/Latino	10.87 (0.00,83.63)	13.6
White	% of population that self-identifies as White, non-Hispanic/Latino	44.83 (0.00, 96.43)	31.77
Hispanic/Latino	% of population that self-identifies as Hispanic/Latino (of any race category)	16.68 (0.00–66.44%)	15.51

1. All independent variable data sourced from the 2013 American Community Survey (ACS).

2. If median income was listed as “250,000+” we assumed a med. income of 250,000.

3. Based on estimates from land-use regression model developed by Simon et al. (2018).

### Statistical analyses

We created maps that displayed UFP concentrations and each demographic indicator using ArcMap 10.5.1. We limited block groups to those located within 1000 m of the mobile monitoring route. Demographic variables were stratified and mapped according to quintiles. We examined correlations between and among socio-demographic variables and UFP concentration using Pearson correlations. Variables with statistically significant correlations below 0.6 were considered for inclusion in subsequent models (Table 1). We built a multivariate linear regression model using stepwise selection including those demographic variables that were statistically significant in univariate models. If two variables were highly correlated with eachother (r>0.75) we included the one that had the greatest predictive value in univariate models with UFP as the outcome (See S1 Table).

We evaluated autocorrelation in our model using the Local Moran’s I cluster and outlier analysis. The Global Moran’s I test provides a value between -1 and 1, with values near 1 indicating clustering of high values near one another while negative values indicate lower values clustering. While there were significantly clustered values in our data only the UFP data was clustered with a score above 0.5 and was predominantly higher in areas closer to major roads (S2 Table). The results did not show a need for alternative regression modeling. All statistical analyses were done using SAS software Version 9.4 of the SAS program for Windows (SAS Institute Inc., Cary, NC, USA).

## Results

### Demographic indicators

After excluding block groups that were located more than 1000 m from the mobile monitoring route or outside Boston, the study area was comprised of 250 census block groups. We also excluded outliers that fell more than 1.5 IQR away from Q3 or Q1 per Tukey’s approach and a very small number of block groups with zero population. Demographic indicators and mean values across the block groups are presented in Table 1. Across all block groups the mean percentage of racial/ethnic minorities was 55.3% (SD: 31.2%), which broke down to 23.1% Black (SD: 25.2%), 10.9% Asian (SD: 13.6%) and 16.7% Hispanic/Latino (SD: 15.5%). Whites comprised 44.8% (SD: 31.8%) of the population. At the block-group level, on average 26.6% of households (SD: 17.8%) lived below the poverty line and the median income was $57,000 (SD: $46,660). Unemployment among adult males was 22.7% (SD: 15.4%) and 12.7% (SD: 13.9%) of households were headed by females. Thirty percent (SD: 21.7%) of households owned their homes.

### UFP concentrations

**Fig 1:**
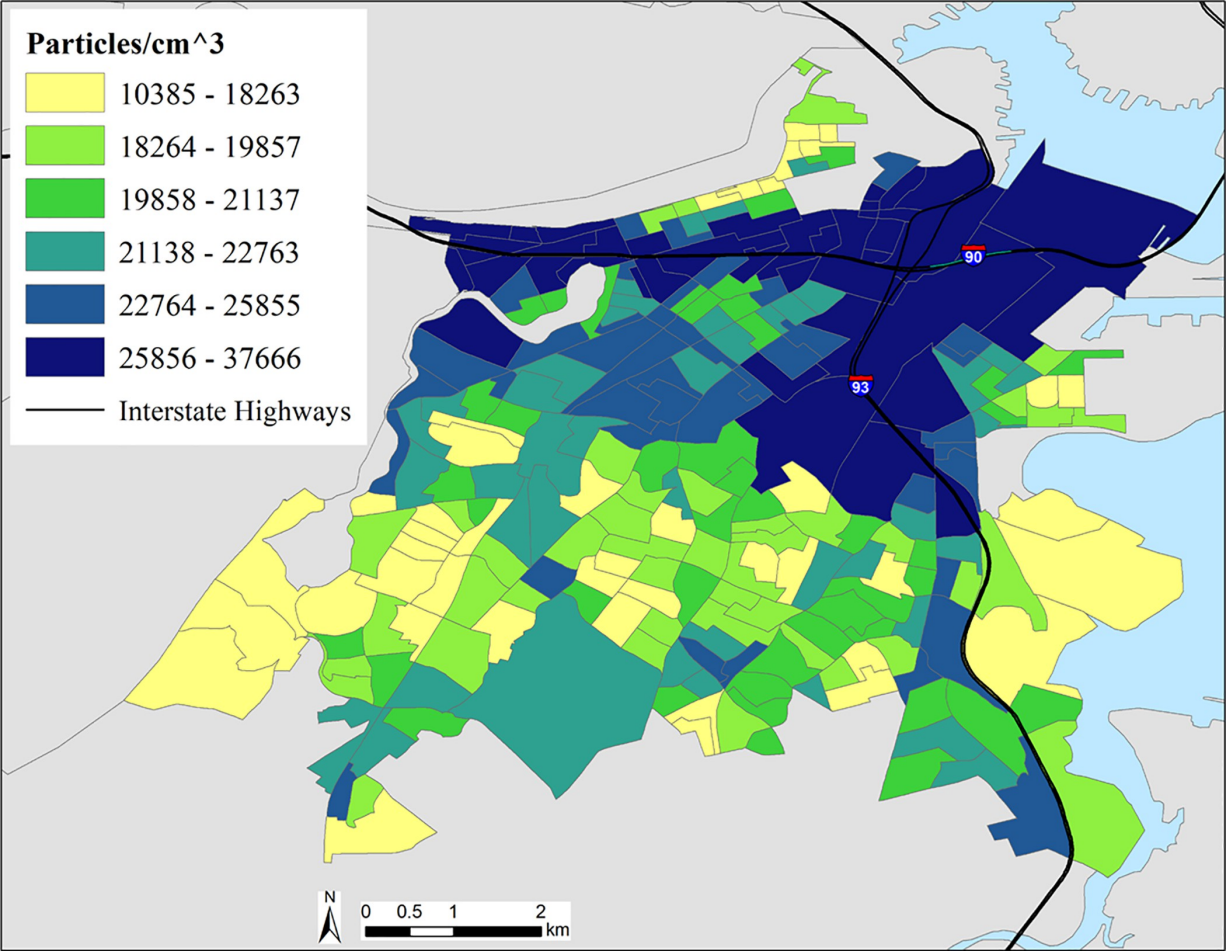
Spatial distribution of UFP concentrations at the block-group level in Boston, MA.

UFP concentrations were not normally distributed, therefore, we log-transformed the data to approximate normality. The highest modeled UFP levels were near and along major roadways and bus routes, and were lower in and near residential areas and open spaces. Mean annual UFP across all census tracts was 25,000 particles/cm^3^; the range of UFP levels by census block group was from 10,385 to 37,666 particles/cm^3^ (SD: 5000) (See Table 1). Fig 1 shows modeled UFP number concentrations by block group. Generally, UFP concentrations were highest in the northern portion of the study area, corresponding to the busiest areas of downtown Boston and the convergence of I-90 and I-93.

### Association between UFP and demographic variables

Pearson correlations of the relationship between demographic indicators and UFP are provided in Table 2. Significant correlations were identified for below poverty, home ownership, median income, female HOH and race/ethnicity. Specifically, there was a significant, but small, 0.20 positive correlation between UFP and poverty level (p< 0.01) and inverse relationship between UFP and homeownership (-0.23). The highest correlation between race and UFP was the 0.41 correlation with predominately Asian block groups. Next was a correlation of -0.27 with predominately Black block groups.

**Table 2:** Pearson correlations of SES indicators with average annual UFP exposure (2013) in Boston block groups within 1000 m of mobile monitoring route^1^.

SES Indicator	Pearson Correlation	
	*correlation coefficient (r)*	*p*
Below Poverty	**0.20**	**< 0.01**
Male Unemployment	0.02	0.71
Education	-0.05	0.43
Public Assistance	-0.09	0.29
Home Ownership	**-0.23**	**< 0.01**
Median Income	**-0.15**	**0.02**
Female Headed Households	**-0.17**	**0.02**
Race/Ethnicity		
Black	**-0.27**	**<0.01**
Asian	**0.41**	**<0.01**
White	**0.13**	**0.04**
Hispanic/Latino	**-0.17**	**<0.01**

^1.^ Outliers and block groups with 0 values excluded.

**Fig 2:**
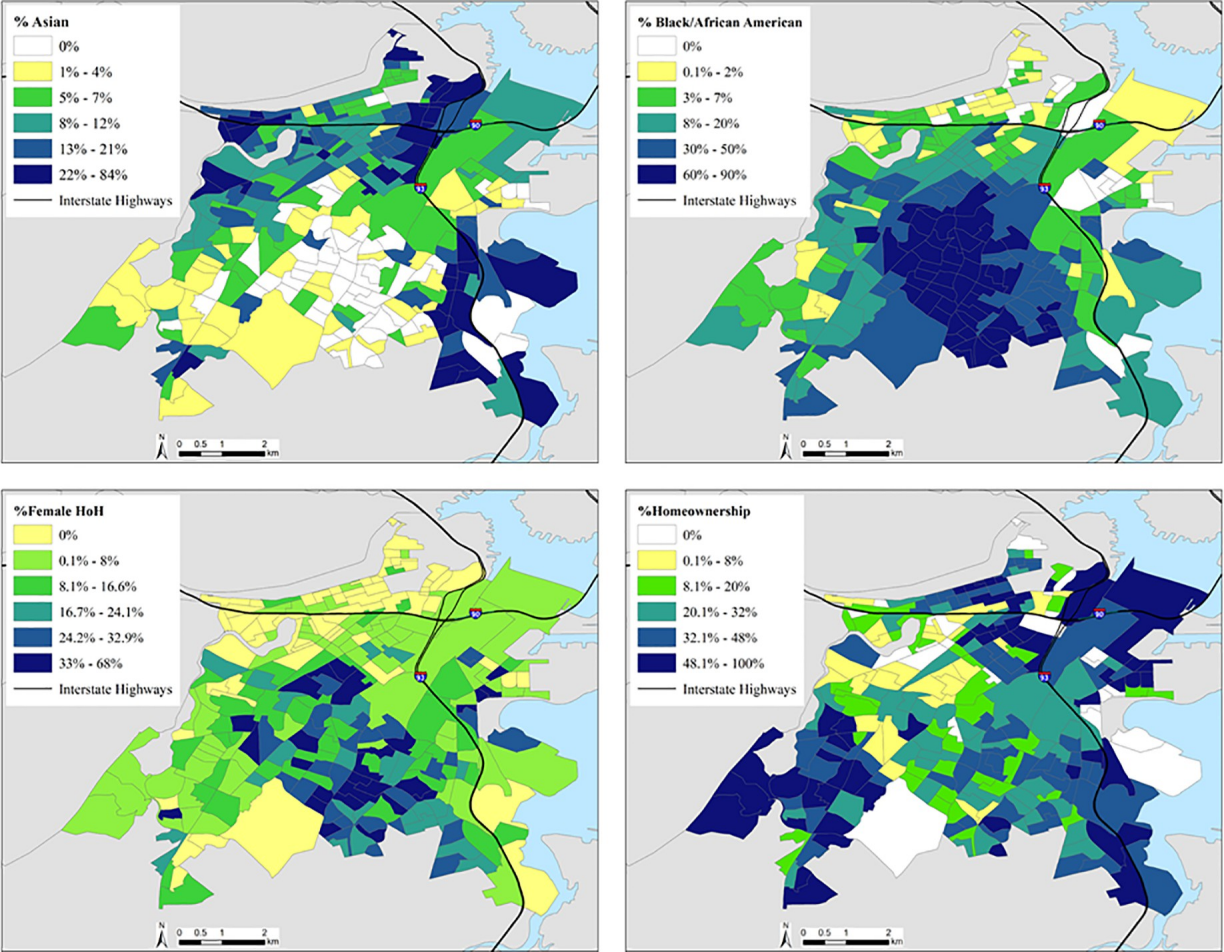
Map of block group level concentrations of variables in the stepwise linear regression model divided into quintiles.

We built a multivariate linear regression model that included significant variables based on stepwise selection. We present the model that best explained variation in UFP concentrations in Table 3. This model includes minority population broken down by Black and Asian race, female HOH, and home ownership. It explains 25.9% of the variation in UFP concentrations. The results show that an increase in Asian population was associated with a statistically significant increase in UFP exposure. Female HOH and homeownership were each associated with statistically significant decreases exposure to UFP. An increase in the Black population was no longer associated with UFP concentrations in the multivariate model. Fig 2 displays maps of the spatial distribution of the four indicators retained in the final multivariate linear regression model: Asian population, Black population, female HOH and homeownership.

**Table 3:** Stepwise-selected regression model of the impact of census block group demographic and socioeconomic variables on ambient UFP number concentrations.

Variable included in model after selection	Slope (SE)*	p-value
Minority population (Asian)	8176.87 (2120.79)	<0.01
Minority population (Black)	-2240.22 (1278.46)	0.08
Home Ownership	-5659.98 (1246.63)	<0.01
Female Head of Household	-4972.40 (2274.72)	0.03

*Slopes reflect change in UFP per every 1% increase in independent variable (per every $10,000 of median income).

## Discussion

Our findings show that block group-level indicators of race/ethnicity and SES were related to the distribution of UFP concentrations in the city of Boston. Specifically, block groups with higher Asian populations were associated with higher UFP concentrations compared to block groups predominated by Whites or other minority groups. In a separate study of pollutants not evaluated here, higher concentrations of air toxics (carcinogenic air pollutants) in areas with higher Asian populations was noted [28]. In addition, we found that in our sample, higher homeownership (indicating higher SES) was associated with lower UFP concentrations. These results are in alignment with other studies identifying higher levels of other air pollutants including PM_10_, NO_2_ and ozone. [22,29]. However, higher female HOH (indicating lower SES) was associated with lower UFP concentrations in our analysis.

The downtown core of Boston had the highest UFP concentrations compared to most other areas of Boston. This is consistent with UFP models in other cities, such as Toronto and Montreal, in which UFP concentrations were elevated in downtown areas compared to those away from the city center [30–32]. Downtown Boston is the part of our study area with the greatest density of transportation infrastructure, including two major highways, bus routes, local street traffic and railroad stations and lines (both commuter and freight). Modelled UFP concentrations were higher near major roadways and bus routes as well as industrial compared to residential areas in prior analyses of Boston [7,24,33]. Similar associations have been found in other cities across the globe [4]. Historically, areas next to highways and busy roads are disproportionately lower SES and higher minority populations, potentially increasing their exposures compared to White populations [11]. Boston’s Chinatown, with a high Asian population, is in the downtown area, which results in a higher potential exposure for this minority group.

From an environmental justice standpoint our results show evidence of inequitable distribution of UFP concentrations based on Asian race and some indicators of SES. These findings may differ from the associations for other pollutants due to the highly localized distribution of UFP in the study area. However, there have been shifts in demographics of race and income in recent years in Boston. At the time of our study there remained many low-income households in and near the downtown core of Boston a large proportion of whom were Asian. However, there has been substantial growth in high-income housing in Chinatown and the larger Downtown Area in recent decades [34]. Over time, the downtown core has become more populated with people that are higher income and White, compared to other areas of the city.

In this sense, Boston mirrors, in a smaller way, the concentration of high-income housing coincident with high pollution levels in, for example, Manhattan [35]. Thus, results suggest that in Boston, high UFP concentrations is associated with demographics in complex ways. A full explanation of the results from the multiple racial/ethnic and SES indicators requires a more thorough examination of the local built and social environments at finer geographic scale than was possible here due to use of block group-level census data.

There are numerous strengths as well as some limitations to our analysis. A key strength was the availability of a fine-grain UFP model built from local mobile and stationary measurements to assign concentrations to census block groups. Although UFP models are increasingly common, they are still rare relative to other air pollutants such as PM_2.5_ and NO_2_ that usually have resolution at a much larger scale. Another strength was that, to our knowledge, there are no prior socio-demographic analyses of UFP, making exploratory work a valuable contribution to the literature and one that should prompt additional research.

Exclusions made were consistent with standard statistical proactice and did not affect the validity of the analysis. The main limitation of our analysis is that the demographic and SES data are at a lower geographic resolution than the UFP model. We were restricted to Census block groups in this analysis because that was the resolution of the American Community Survey data that is publically available. Multiple monitoring campaigns and two models of UFP in the Boston area, including the one we used, consistently show gradients of UFP within 100–200 m of highways and major roadways [24,36]. These gradients are much smaller than the majority of census block groups, and therefore, their effect would not be visible. Thus, finer grain demographic/SESdata or individual data may reveal additional associations with UFP levels.

There is a need for additional analysis of other geographic areas as well as broader geographic regions. While our findings can probably be generalized to the entire city of Boston and the Boston Metropolitan Area, they may not be representative of other urban areas, especially outside of the Northeastern US.

## Conclusions

To our knowledge, our study is the first to examine associations between UFP concentrations and socio-demographic indicators. We found that race and socioeconomic indicators were associated with variations in UFP levels. Given the high spatial variation of UFP and dense population within the City of Boston, this potentially puts large numbers of people at risk for high exposures. Our findings show the importance of evaluating the local built environment and its relationship to air pollution, race and socioeconomic indicators. Additional studies in other major cities using spatially resolved data are necessary to determine the importance of local factors on UFP exposure.

## Supporting information

S1 Fig SF1: Map of Boston neighborhoods. (TIF)

S1 Table Pearson correlation coefficients between All SES variables examined for model inclusion. (DOCX)

S2 Table Global Moran’s I cluster test (all cluster were significant below p-value 0.01). (DOCX)
