# Supplementary figures and images for: An exploratory analysis of sociodemographic characteristics with ultrafine particle concentrations in Boston, MA
Source: PLoS One. 2022 Mar 30;17(3):e0263434. doi: 10.1371/journal.pone.0263434 (PMC8967040; doi:10.1371/journal.pone.0263434)

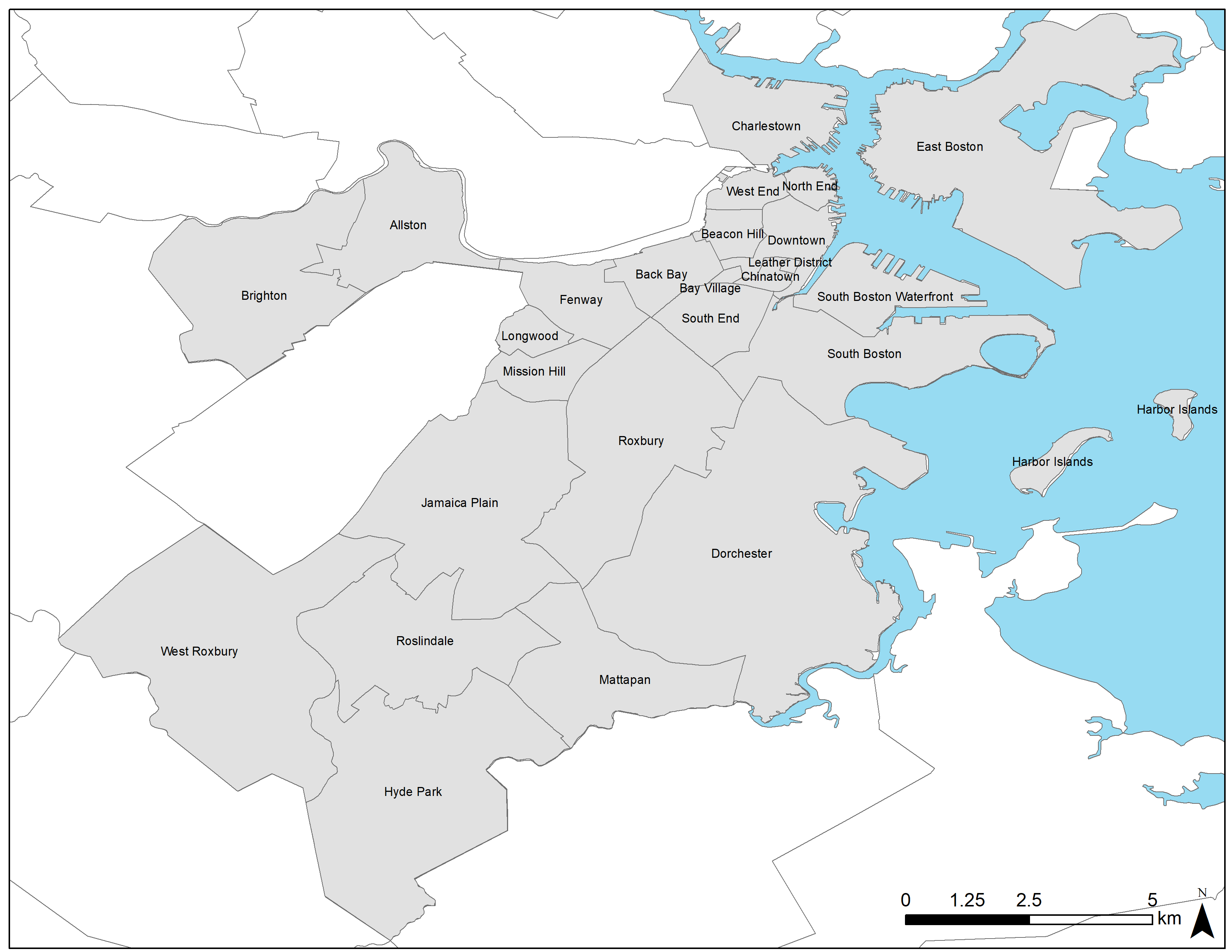

Supplement: S1 Fig — (TIF) [file pone.0263434.s001.tif]
