# Supplementary material for: An exploratory analysis of sociodemographic characteristics with ultrafine particle concentrations in Boston, MA
Source: PLoS One. 2022 Mar 30;17(3):e0263434. doi: 10.1371/journal.pone.0263434 (PMC8967040; doi:10.1371/journal.pone.0263434)
Supplement: S1 Table — (DOCX) [file pone.0263434.s002.docx]

| **S1 Table: Pearson Correlation Coefficients between All SES Variables Examined for Model Inclusion** | | | | | | | |  |  |
| --- | --- | --- | --- | --- | --- | --- | --- | --- | --- |
|  | Median Income | % Employed Working Aged Males | % High School Educated | % Receiving Public Assistance | % Below Poverty Line | % of Households with Female Heads and Children < 18 y/o | % Homeownership |  | Cell Legend |
| Median Income | 1.00 | 0.55 | -0.51 | -0.38 | -0.75 | -0.45 | 0.74 |  | Pearson's Coefficient |
|  |  | <.0001 | <.0001 | <.0001 | <.0001 | <.0001 | <.0001 |  | p-Value |
|  | 247 | 247 | 247 | 247 | 247 | 247 | 247 |  | N |
| % Employed Working Aged Males |  | 1.00 | -0.47 | -0.39 | -0.56 | -0.45 | 0.42 |  |  |
|  |  |  | <.0001 | <.0001 | <.0001 | <.0001 | <.0001 |  |  |
|  |  | 250 | 250 | 249 | 249 | 249 | 249 |  |  |
| % High School Educated |  |  | 1.00 | 0.54 | 0.43 | 0.58 | -0.39 |  |  |
|  |  |  |  | <.0001 | <.0001 | <.0001 | <.0001 |  |  |
|  |  |  | 250 | 249 | 249 | 249 | 249 |  |  |
| % Receiving Public Assistance |  |  |  | 1.00 | 0.31 | 0.67 | -0.26 |  |  |
|  |  |  |  |  | <.0001 | <.0001 | <.0001 |  |  |
|  |  |  |  | 249 | 249 | 249 | 249 |  |  |
| % Below Poverty Line |  |  |  |  | 1.00 | 0.37 | -0.72 |  |  |
|  |  |  |  |  |  | <.0001 | <.0001 |  |  |
|  |  |  |  |  | 249 | 249 | 249 |  |  |
| % of Households with Female Heads and Children < 18 y/o |  |  |  |  |  | 1.00 | -0.27 |  |  |
|  |  |  |  |  |  |  | <.0001 |  |  |
|  |  |  |  |  |  | 249 | 249 |  |  |
| % Homeownership |  |  |  |  |  |  | 1.00 |  |  |
|  |  |  |  |  |  |  |  |  |  |
|  |  |  |  |  |  |  | 249 |  |  |
