# Supplementary material for: An exploratory analysis of sociodemographic characteristics with ultrafine particle concentrations in Boston, MA
Source: PLoS One. 2022 Mar 30;17(3):e0263434. doi: 10.1371/journal.pone.0263434 (PMC8967040; doi:10.1371/journal.pone.0263434)
Supplement: S2 Table — (DOCX) [file pone.0263434.s003.docx]

S2 Table: Global Moran's I cluster test (all cluster were significant below p-value 0.01)

| Variable | Global Moran's I |
| --- | --- |
| Particle Number Concentration | 0.67 |
| Median Household Income | 0.41 |
| % Below Poverty | 0.34 |
| % On Public Assistance | 0.25 |
| % Owner Occupied Households | 0.42 |
| % Female Head of Household | 0.44 |
| % Male Unemployed | 0.14 |
